# MnemoCity Task: Assessment of Childrens Spatial Memory Using Stereoscopy and Virtual Environments

**DOI:** 10.1371/journal.pone.0161858

**Published:** 2016-08-31

**Authors:** David Rodríguez-Andrés, M.-Carmen Juan, Magdalena Méndez-López, Elena Pérez-Hernández, Javier Lluch

**Affiliations:** 1 Instituto Universitario de Automática e Informática Industrial, Universitat Politècnica de València, Camino de Vera, s/n. 46022, València, Spain; 2 Departamento de Psicología y Sociología, Universidad de Zaragoza, Zaragoza, Spain; 3 Departamento de Psicología Evolutiva y de la Educación, Universidad Autónoma de Madrid, Madrid, Spain; Universiteit Utrecht, NETHERLANDS

## Abstract

This paper presents the MnemoCity task, which is a 3D application that introduces the user into a totally 3D virtual environment to evaluate spatial short-term memory. A study has been carried out to validate the MnemoCity task for the assessment of spatial short-term memory in children, by comparing the children’s performance in the developed task with current approaches. A total of 160 children participated in the study. The task incorporates two types of interaction: one based on standard interaction and another one based on natural interaction involving physical movement by the user. There were no statistically significant differences in the results of the task using the two types of interaction. Furthermore, statistically significant differences were not found in relation to gender. The correlations between scores were obtained using the MnemoCity task and a traditional procedure for assessing spatial short-term memory. Those results revealed that the type of interaction used did not affect the performance of children in the MnemoCity task.

## 1. Introduction

One of the most critical cognitive abilities in humans is storing the representation of stimuli that were experienced at a certain time in the past. Hence, memory can be divided into short-term and long-term, depending on whether the memory formed has a limited time period or a longer and stable one [1]. Spatial memory generally refers to the ability to store representations of spatial stimuli. This type of memory allows us to find a place that was visited previously, follow a route after consulting a map or remember the place where we left our belongings, among other examples [2].

The use of computer-based technologies has increased in a variety of fields and may provide an advantage over traditional methods. This has already been demonstrated in fields like psychology or education [3–7]. In our work, the advances in these computer-based technologies have been used to develop an application to evaluate spatial short-term memory. Our application introduces the user into a virtual environment. The stereoscopy technology gives the user a greater sense of immersion. Our application was created in the field of psychological assessment, which is focused on testing a human cognitive ability, spatial short-term memory, from an ecological assessment perspective.

The use of virtual reality to assess spatial memory in humans has shown positive results [8–11]. There are systems created for evaluating spatial memory in humans. Most of them are based on tasks that have been previously used in animal research [10–12]. These systems introduce the user into a virtual environment, where the user can move and interact with the systems. However, the systems developed for humans used to include classical interactions (e.g., a computer screen, a mouse, or a keyboard). These systems were designed to assess spatial memory in adults. Our application was especially designed to assess spatial memory in children. Hence, the duration of the task, the type of stimuli, and the reinforcements used take this population into account. In addition, the human-computer interface of our system consists of a 120" stereoscopic screen and a Natural User Interface (NUI) to facilitate the children's interaction with the system, and to improve the immersion in the virtual environment. The objective of this application is to remember the spatial locations where objects have been previously presented.

To demonstrate the relation between the performance in this novel task and the performance on other classical pencil-paper tests which are commonly used to test spatial short-term memory, the children performed classical tests to assess spatial short-term memory. Therefore, correlations between the results on classical pencil-paper tests and the results of the application can be verified.

The main hypothesis is that there would be statistically significant differences for the score obtained in the MnemoCity task using natural interaction when compared with standard interaction. There are two sub-hypotheses derived from the main hypothesis. The first sub-hypothesis is that there would be no statistically significant difference for the performance of the task between genders. The second sub-hypothesis is that the preference of the user would be the natural user interface. The secondary hypothesis of this work is that the MnemoCity task can evaluate short-term spatial memory in children like the pencil-paper tests applied in psychology.

Section 2 introduces the state of the art for virtual environments and short-term memory evaluation. Section 3 describes the development of the system. Section 4 explains the procedure for testing. Section 5 presents the results and Section 6 presents the discussion. Section 7 presents our conclusions.

## 2. Background

This section introduces virtual environments, Natural User Interfaces, and stereoscopy. We also describe how computer-based technologies have been previously used for the assessment of short-term memory.

### 2.1. Virtual environments

New developments in virtual reality allow new applications for humans to be created. A virtual environment simulates physical presence in places in the real world or in imagined worlds and lets the user interact in that world. Virtual reality artificially creates sensory experiences, which can include sight, hearing, touch, smell, and taste. The benefits of using virtual environments (VEs) in psychology arise from the fact that movements in virtual space and accompanying perceptual changes are treated by the brain in much the same way as those in an equivalent real space [13].

VEs could be a great tool for specific areas like psychology [14]. In this area, the VEs are used to help with specific problems. For example, Holden [15] did a survey of the virtual environment for motor rehabilitation. The benefits of VEs for children with disabilities were studied in [16,17]. Hamilton et al. [18] adapted a virtual adaptation of a behavioral paradigm for the study of spatial memory in rodents to be used for humans. The virtual scene was a circular pool inside a room with four walls. The user had to swing and observe different objects that appear on the walls (cues). Another virtual environment was developed by Moffat et al. [19]. In their study, the males were better than the females at using their egocentric orientation skill in terms of accuracy and speed. Cánovas et al. [10] developed an application to study the effectiveness of a new virtual task to evaluate spatial learning in adults. The system was called the Boxes Room and the design of the task was based on the hole-board. This is a task that is well known in animal research in which the holes to be remembered by the rodent are rewarded with a pellet [20,21]. When translated into a virtual reality environment, the boxes that were used had to be opened to discover a possible reward inside. Cánovas et al. [22] also carried out a study to examine the influence of the number of cues and their location in adult spatial learning for the same task. The study by Koenig et al.’s [8] proposes a spatial memory task with high ecological validity that can be integrated into any virtual environment. Environments and target objects can be individually designed for each user in order to provide a relevant context and high motivation for patients with cognitive deficits. Sturz et al. [23] developed an application to evaluate spatial memory in adults using the valve engine. The results of their study provide empirical evidence for the encoding of variability of landmark-based spatial information and have implications for theoretical accounts of spatial learning. Almost all applications that are developed for the assessment of spatial memory are based on squared virtual environments; however, there are other works that have opted for environments with a different geometric shape. For example, Cimadevilla et al. [12] chose a circular environment. With this circular wall, the users could not use the four walls of a squared environment to help their orientation.

In addition to these studies which focus on adult performance, there are other studies that use virtual environments to assess spatial memory in children. For example, Hamilton et al. [24] used a virtual task called the Virtual Morris Water Maze with children. They demonstrated that children with Fetal Alcohol Syndrome have difficulties in spatial short-term memory. Even though all of these works demonstrate the possibility of using the VE to evaluate memory, these virtual environments have not been specifically designed for children, and the interaction with the system is not adapted to them. In addition, these environments are not natural for children and can contain elements that confuse them. For this reason, we have created an environment with familiar objects that children see in their houses.

In summary, the use of a virtual environment offers the possibility of introducing the user into a virtual world which allows the simulated situation of individuals to be assessed in their daily life.

### 2.2. Natural User Interfaces

NUI are defined as interfaces in which a person interacts with the system with his/her body (hands, legs or any other parts of the body). Another characteristic of the NUI is that the learning process is fast, and the user can move from novice to expert in a quick transition. These user interfaces have previously been used in studies to create an interface that is adapted to children obtaining good results [3,25]. There are other studies that analyze the advantages and disadvantages of NUI and compare them with the standard interaction methods [26,27]. Rauterberg [26] carried out a study to compare four different types of interaction: (1) a command language, (2) a mouse, (3) a touch screen, and (4) a custom-made Digital Playing Desk. They used an implemented version of the computer game "Go-bang". The user had to play the game by moving a real chip on the virtual playing field using the four different types of interaction. A total of 304 visitors rated the usability of all four different interactions on a bipolar scale. The touch interaction was rated as being the easiest to use, followed by the mouse, the Digital Playing Desk, and the command language interface. Lacolina et al. [27] compared two interactive systems for natural exploration of 3D models. They compared two natural interfaces: multitouch vs. free-hand gestures. They concluded that both interfaces provide a natural dual-handed interaction and at the same time free the user from the need to use a separate device. The natural interfaces have proven to be useful for certain purposes such as learning [28,29] or rehabilitation [30]. Most systems developed [10,18,19,23] for evaluating spatial short-term memory which are described in the virtual environments section have simple interaction methods (based on a screen, a mouse, and a keyboard).

### 2.3. Stereoscopy

**Fig 1:**
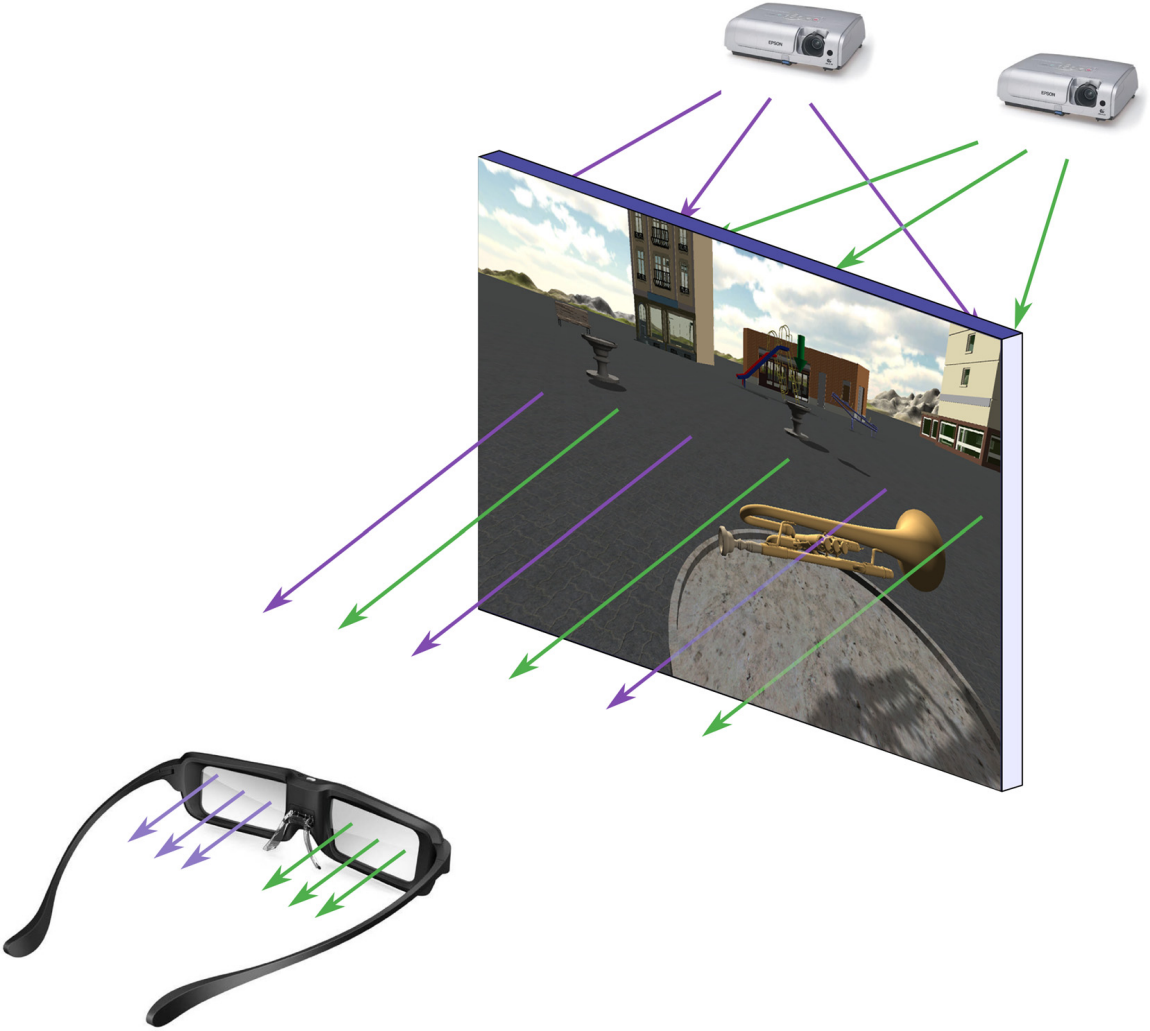
Stereoscopy scheme. The two projectors send polarized images to the screen. The filters placed in the glasses allow each eye to get the correct image.

Stereoscopy is a technology that is currently being incorporated in many fields such as psychology [31] and education [32]. Stereoscopy is based in collecting three-dimensional visual information and creating an illusion of depth. This can be obtained by showing a different image to each eye (Fig 1). In an ideal 3D application, the users perceive how the objects come out of the screen, and they have the illusion that the objects are in front of them. By adding this technology, a virtual environment can be improved by providing an immersive sensation, and making the users feel as if they were actually inside the virtual world [14]. This technique has been used to introduce users into the virtual environment and create the sensation that he/she is in a real place performing a real task. For example, Westwood et al. [33] explained that there are differences between surgeons who were trained with a virtual reality simulator and surgeons that were not trained with it. This virtual task allows the surgeons to improve their skills in a simulated environment. Since it has been demonstrated that stereoscopy increases the immersive sensation of the user in the task, we have selected this technology for memory assessment.

### 2.4. Short-term memory

Spatial memory is a higher cognitive function that has been extensively probed using testing paradigms that were developed for animal models with the aim of understanding the neural basics of memory. Hence, there is a large body of knowledge about how our brain works to store information (i.e., to create memories) that is derived from the data about performance in tasks for spatial training [34]. Spatial tasks of this type can be defined according to the type of memory trained (i.e., short-term or long-term) [1]. Spatial short-term memory is defined as the limited capacity of subjects to remember the locations of items for short periods of time [35]. In children, the spatial short-term memory ability is related to academic outcomes [36]. Therefore, it is interesting to assess this type of memory in children and to obtain information that could predict their academic achievements. Most of the tasks developed to test spatial short-term memory in children consist of showing very simple items or objects on a screen, a paper, or a board (e.g., [37–39]). In these tasks, the person is tested while sitting in a chair; however, spatial memory has a strong component of spatial orientation, which is only tested when the spatial items to be remembered are located in a more complex layout. Spatial orientation involves establishing a relationship between the spatial elements of a large environment, where the person and the spatial items are located. This problem can be solved by using virtual environments, which do not require a large interaction area.

Thanks to advances in virtual reality, several tests have been developed for the assessment of short-term memory for spatial locations [6,10,22]. However, to our knowledge, none of them have used the types of interaction that are used in our task, nor have they used stereoscopic visualization.

## 3. Materials and Methods

In this section, we describe the task that was developed to carry out our study. We also explain in detail how we developed the system and the software and hardware used.

### 3.1. The MnemoCity task

**Fig 2:**
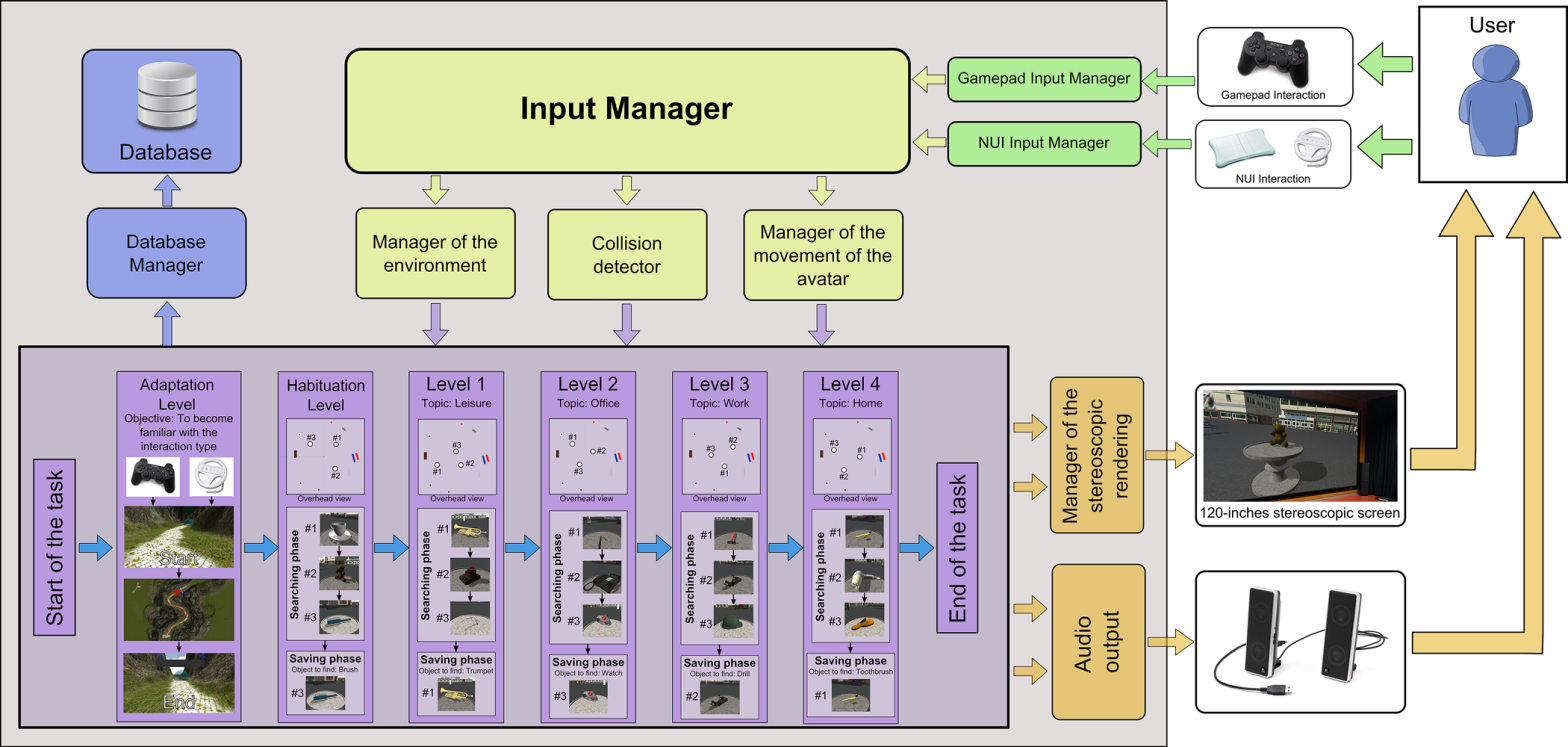
General scheme of MnemoCity task. An explanation of the elements that appear in overhead views can be found in Fig 6.

The main objective of the user in the MnemoCity task is to search for objects and remember their location. Fig 2 shows a general scheme of the MnemoCity task. The objects appear in the virtual environment. The MnemoCity task has six levels: an interaction adaptation level, a habituation level, and four levels for the main task. The objective of each level of the task is described below.

- The interaction adaptation level: This level aims to provide an initial experience with the system so that the user becomes familiar with the interaction for the main task. In this level, the user learns how to move inside the virtual environment using one of the two types of interactions.
- The habituation level: This level is the tutorial level of the main task. In this level, the user learns what the goal of the task is and how to achieve it.
- The main task: This task is composed of four different levels. The goal of these levels is to assess children short-term memory for object location. In this level, the task stores the information about the user’s performance at each level, which reflects the spatial short-term memory ability of the user.

**Fig 3:**
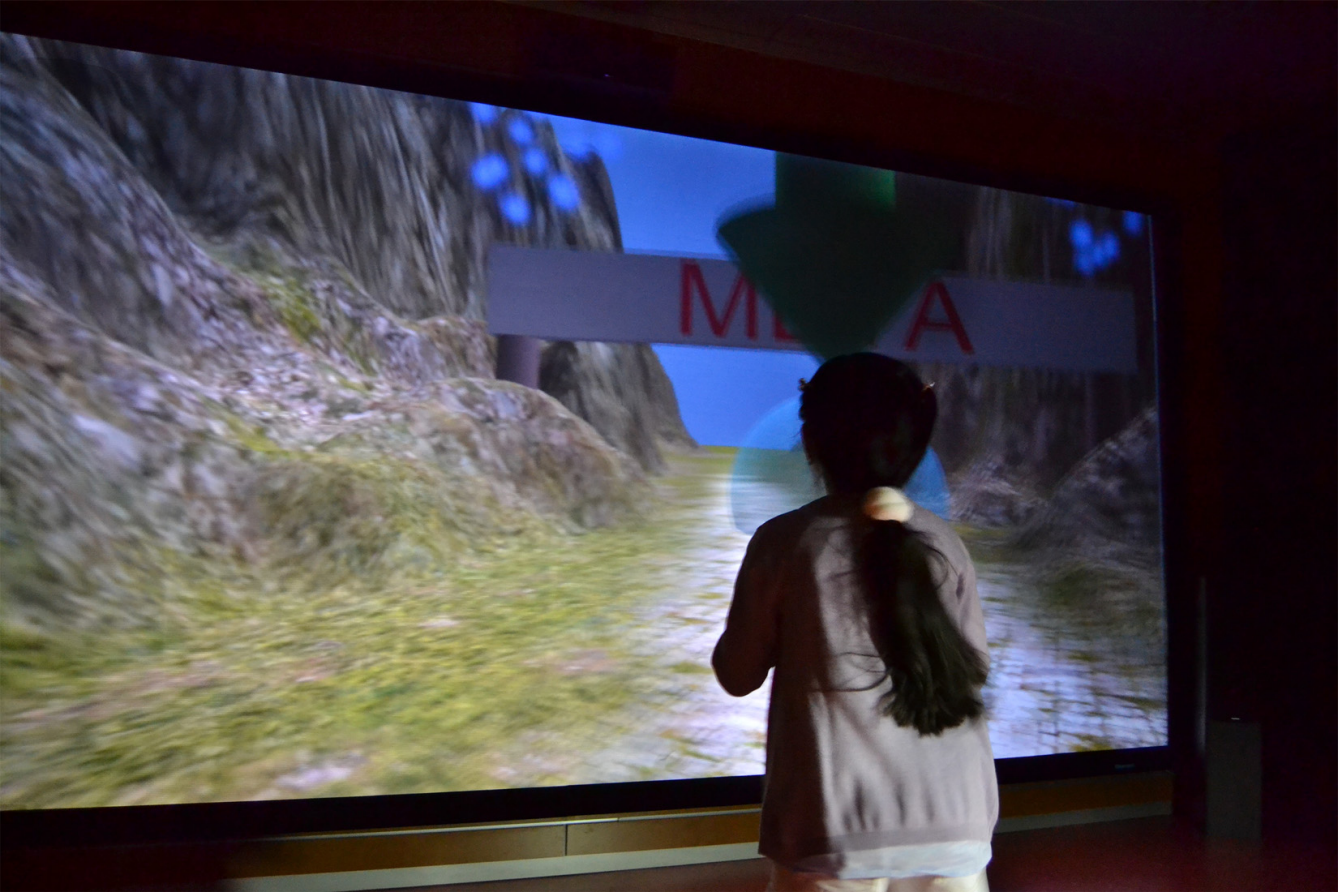
A child performing the interaction adaptation level.

The interaction adaptation level consists of a path through mountains that the users must follow. There are arrows and bubbles along the path that help the user to find the direction to be followed. The path forward has several curves. The aim of these curves is for the user to follow the path as it curves from left to right and get used to the interaction. At the end of the path, there is a big sign that indicates the end of the level. Fig 3 shows a child performing the interaction adaptation level.

After completing the interaction adaptation level, the user is moved virtually to the practice level of the memory task: the habituation level.

In the habituation level and the four levels of the main task, the user is located in a virtual city. This environment consists of a city square that is surrounded by several buildings (distal cues), and eight visual cues inside the city square (proximal cues). Since the geometry of the land may or may not help user orientation [40], our environment was created in a square shape to help the participant navigate. Because the application was designed for children, an open space (city square) was chosen. This is to prevent the children from being in a virtual environment that is too closed or too dark and could frighten them. The visual cues help the user to orientate spatially.

The habituation level is similar to the rest of the levels of the main task. This level was designed so that the users could learn how to perform the task. Throughout all of the levels, a narrator guides the children with her voice and tells them what to do each time (e.g., *“You have to put this object in its correct position”*, “*Remember the location of the objects that you are going to see now”*,*”Approach the table and push the button when it changes color”)*.

Each level is divided into two phases. In the first phase, called the searching phase, the users must move through the environment looking for a green arrow. This arrow is pointing to a white table. The child must walk to this table, and when the child is close enough, the table changes color from white to green, and the child can see the secret object on the table. The child must repeat this process two more times to discover a total of three objects. It is important to note that the children must remember the objects they saw and where the objects were placed. At the end of each search phase, the child returns to the center of the scene, and the virtual world is rotated 180° from the original position before starting the second phase. Therefore, the idiothetic information cannot be used as a reference for orientation.

In the second phase, the saving phase, the screen shows an object and the narrator asks the user the position of that object. From a cognitive perspective, the searching phase refers to the formation of short-term memories for visuospatial items, whereas the saving phase refers to the retrieval of those items.

**Fig 4:**
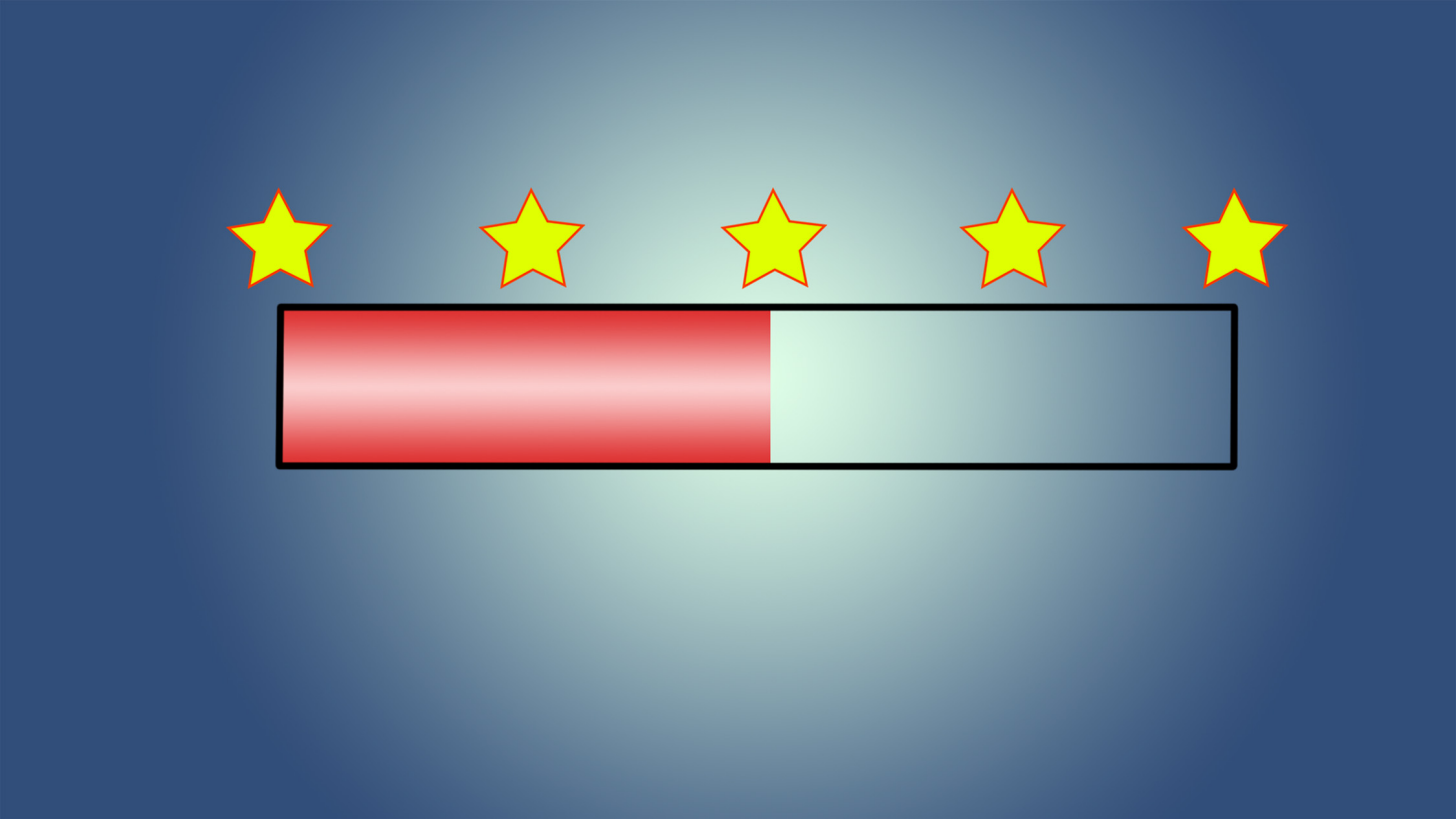
The progression bar with the five stars needed to complete the game.

**Fig 5:**
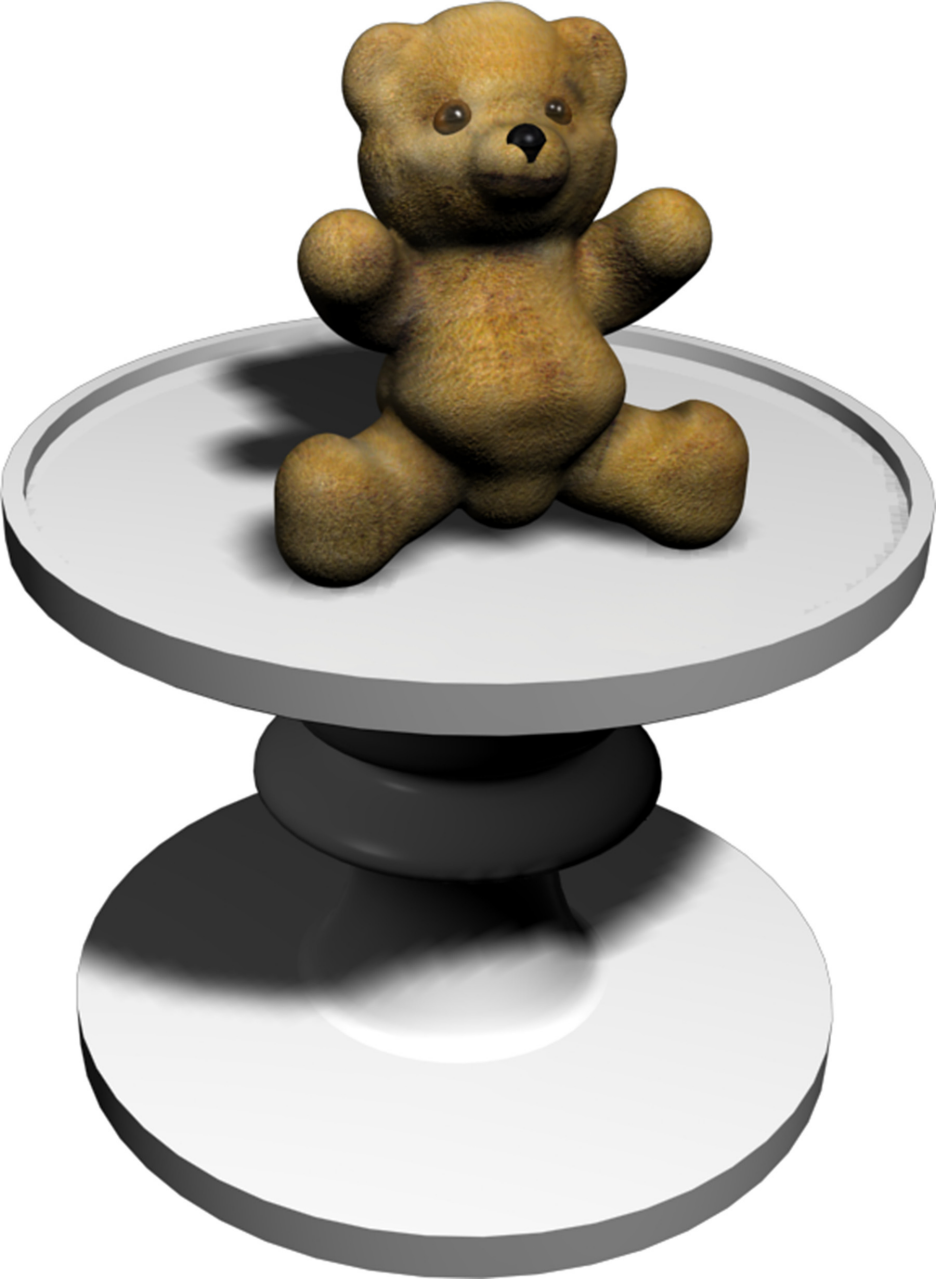
An example of one of the objects that appears on the table.

To keep the child motivated, the game includes a score screen. The child receives a star when he/she finishes the habituation level and the four levels of the main task, regardless of the quality of his/her responses. Hence, when the task is completed, the user has obtained five stars, which are shown on the score screen (Fig 4). From the perspective of the user, it does not matter whether or not he/she places the object on the correct table because, in all cases, the user goes on to the next level. This is to keep the child from becoming frustrated with an incorrect response that could affect another level. However, the selected object is stored in the database. Each level has three tables and three hidden objects to remember. To complete the game the participant must complete the interaction adaptation level, the habituation level, and the four levels of the main task. When the child is in the virtual environment of the city, all of the hidden objects of one level have a common theme. The objects and the theme of each level are the following: Habituation level: a coffee cup, a teddy bear (the object shown in Fig 5), and a brush; Level 1 (Leisure level): a trumpet, a camera, and glasses; Level 2 (Office level): a pen, a telephone, and a watch; Level 3 (Work level): a hammer, a drill, and a helmet; and Level 4 (Home level): a toothbrush, a hairdryer, and a slipper. The three objects that characterize each level and the object that the child is asked to locate are shown in Fig 2.

### 3.2. Development

**Fig 6:**
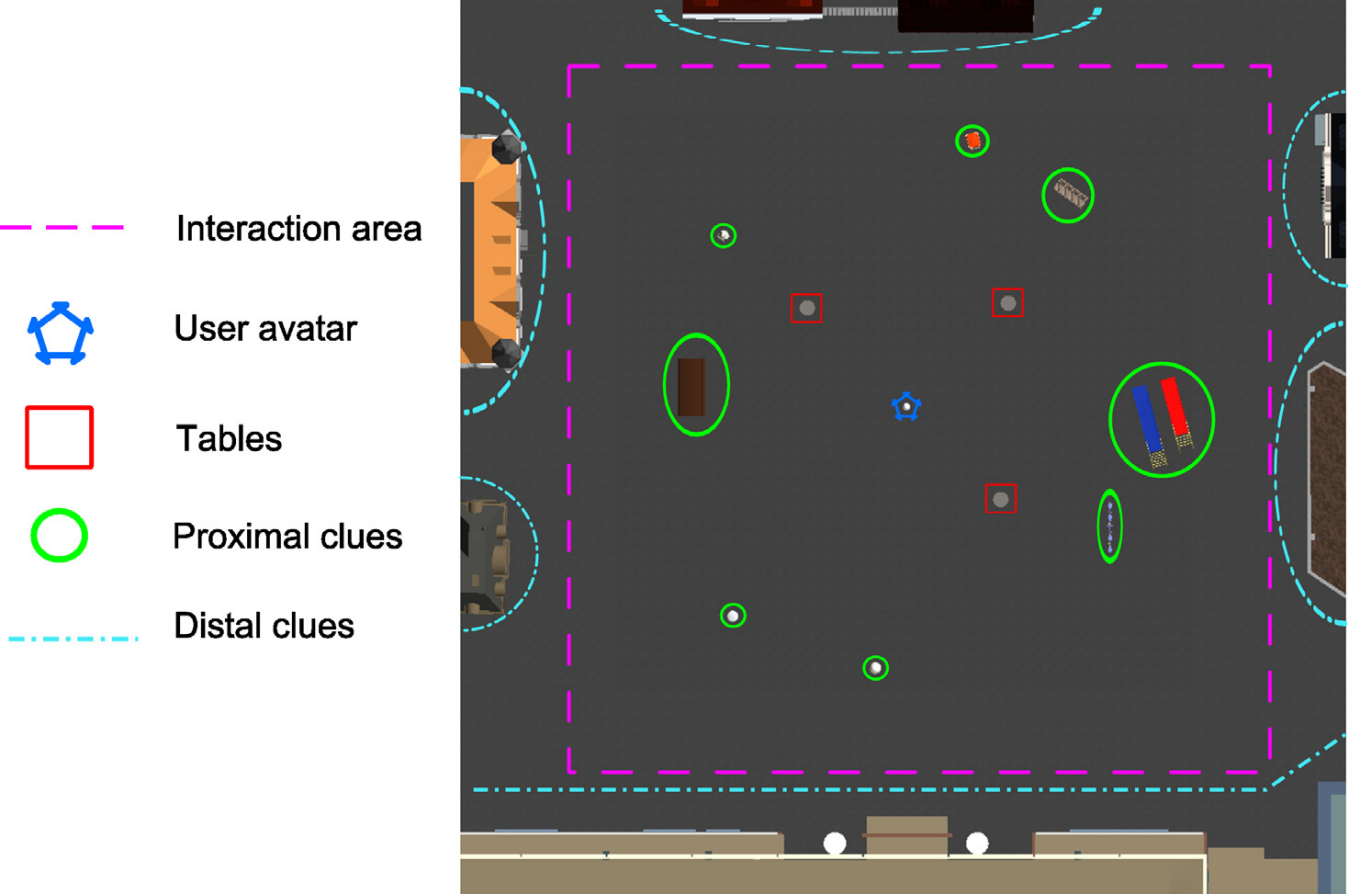
The virtual environment as seen from above.

The system is divided into three main components: the passive environment, the active environment and the user interaction. The passive environment was developed first. This passive environment consists of the objects that are static in the virtual world (e.g., buildings, the ground or the bench). To create all of these objects, we used a 3D model library called *De Espona*. These models were improved by using Blender and 3DS MAX to adapt the characteristics to the application requirements. The passive environment is composed by a city square surrounded by 16 visual cues including 8 buildings and 8 objects that are commonly found in a city (a streetlight, a bench, a trash can, a statue, a bin, a bus stop, a swing and a slide). The buildings are located distally, and the remaining visual cues are placed proximally to the city squared. Fig 6 shows the city square as seen from above.

**Fig 7:**
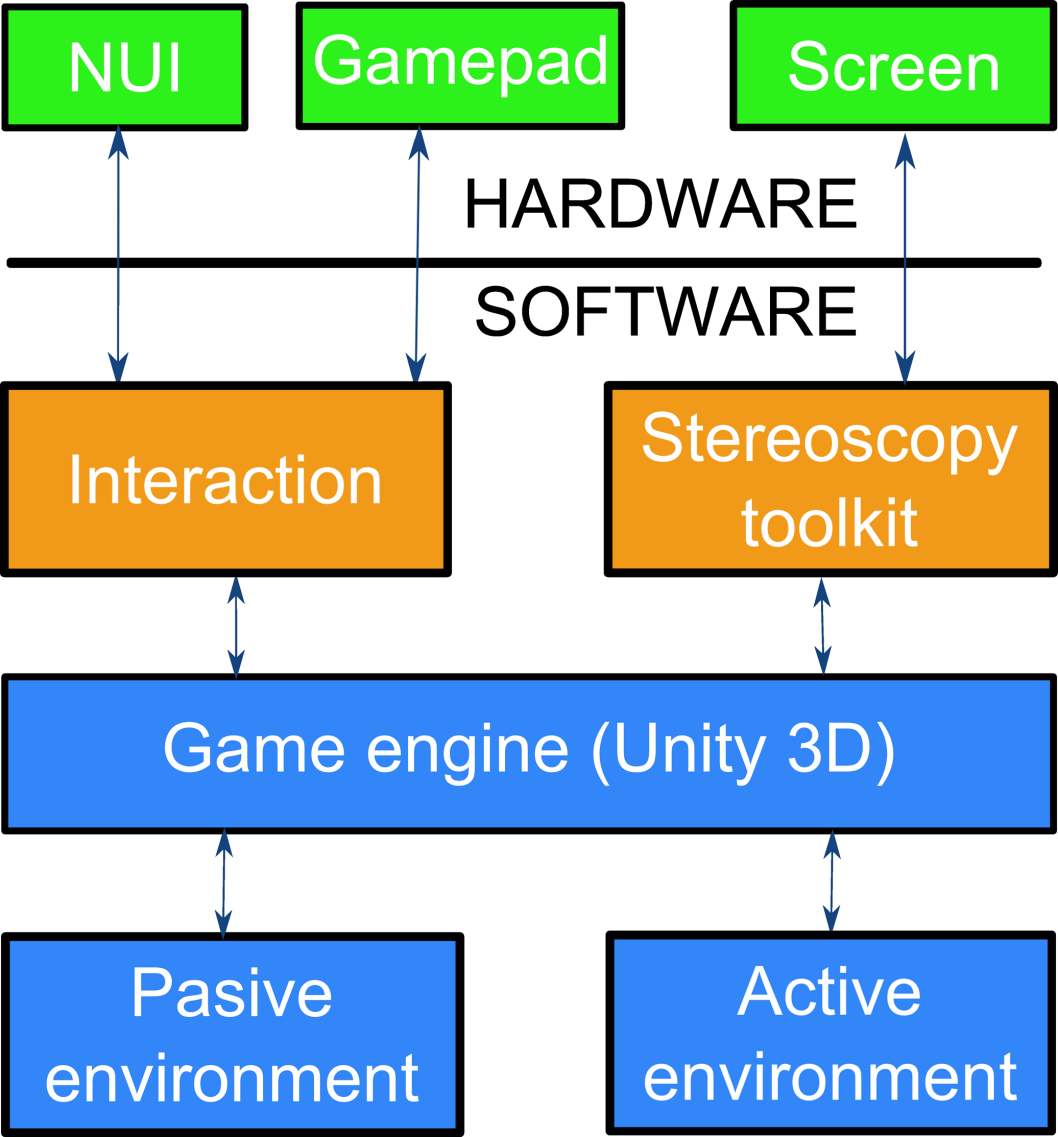
System architecture.

The interaction area of the virtual environment limits the movement of the child (Fig 6 pink dashed lines in front of buildings). These limits are used so that the children will not try to walk inside the buildings and be distracted from the main task. The 16 visual cues have colliders to prevent the child from walking through them and to look more realistic. The active environment has two principal components. The first component is the child’s avatar. This avatar is a representation of the user in the virtual world. The second components are the tables. There are three tables and their position change in every level but the distance between them, and between them and the avatar, is similar in each level. The system includes all the code that allows the visualization for the virtual 3D environment. A library to create the 3D sensation for the children was also developed. This library allows us to place two cameras on the child’s avatar, and each camera simulates one of the eyes of the user. The cameras are located at a standard intraocular distance (63 mm) [41] and at a field of view of 60°. This value for the field of view was calculated from the real dimensions of the screen and the distance between the participant and the screen. We used Unity 3D (http://unity3d.com) as a game engine to merge all of the characteristics of the system into one application. Fig 7 shows the architecture of the system.

The immersive property of our system is based on the fact that the user can see the objects of the virtual environments come out of the screen as if they were real objects, creating the feeling that the user really thinks that he/she is inside the virtual environment and he/she is walking around it. The 120-inch screen acts as a window of union between the real and the virtual worlds, and the stereoscopy creates the illusion that the virtual world occupies the real world where the user is. Thus, the system creates the feeling of immersion for the user.

### 3.3. Interaction

One of the two types of interaction is performed using NUI. The user moves in the virtual environment by physically walking on a Wii Balance Board. A change in direction is achieved by turning a wireless steering wheel. To compare this type of interaction with a more standard type of interaction without physical movement, we use a gamepad. These two interfaces were developed to facilitate user interaction in the application.

- NUI interaction: Wii mote and Wii balance board interaction were used. The user is placed on the Wii balance board and he/she holds a Wii mote that looks like a steering wheel. When the user raises his/her foot the avatar starts to walk. These movements were designed to be as intuitive as possible. The user can turn left or right using the steering wheel to complete the mobility of the avatar. With these two movements, the child can walk around the scene. The Wii mote has an action button that the children press when they want to see the object on the tables or to place the object on one of the tables.
- Gamepad interaction: To design a more standard interface that could be used in a seated or standing position, we have taken into account that children are accustomed to using their hands to interact with different devices like video game consoles, computer games with gamepads, smartphones, etc. Therefore, we selected a device that is familiar to them, a PlayStation gamepad. In our system, the movement is controlled with the left joystick of the gamepad (forward, backward, left, and right). The X button of the gamepad is used to see the object on the tables or to place the item on one of the tables.

To play the application, the user must stay in front of the screen and use the interaction device. Since, this application includes passive 3D, the user must wear linear polarized glasses to perceive the 3D sensation. These glasses have two vertical polarizers, one for each eye. There is a difference of 90 degrees between the directions of the two polarizers.

### 3.4. Software and Hardware

We used the following software to develop the MnemoCity task:

- Unity (also called Unity3D) as the game engine. This engine was chosen because it allows the completed application to be developed with the features that we needed. It supports code written in C#, JavaScript, and Boo.
- C# was used to program the scripts in Unity. C# was also used for the creation of an external wrapper that allows us to work with the Wii Mote and the Wii Balance Board.
- Blender and 3DS Max were used to create and improve the 3D models, that are included in the application.
- Adobe Photoshop was used to modify the textures and images.
- The Wiilib3D (http://wiimotelib.codeplex.com) open source library was used to create the application that connected the game with the gamepad, Wii mote, and Wii balance board interactions.

**Fig 8:**
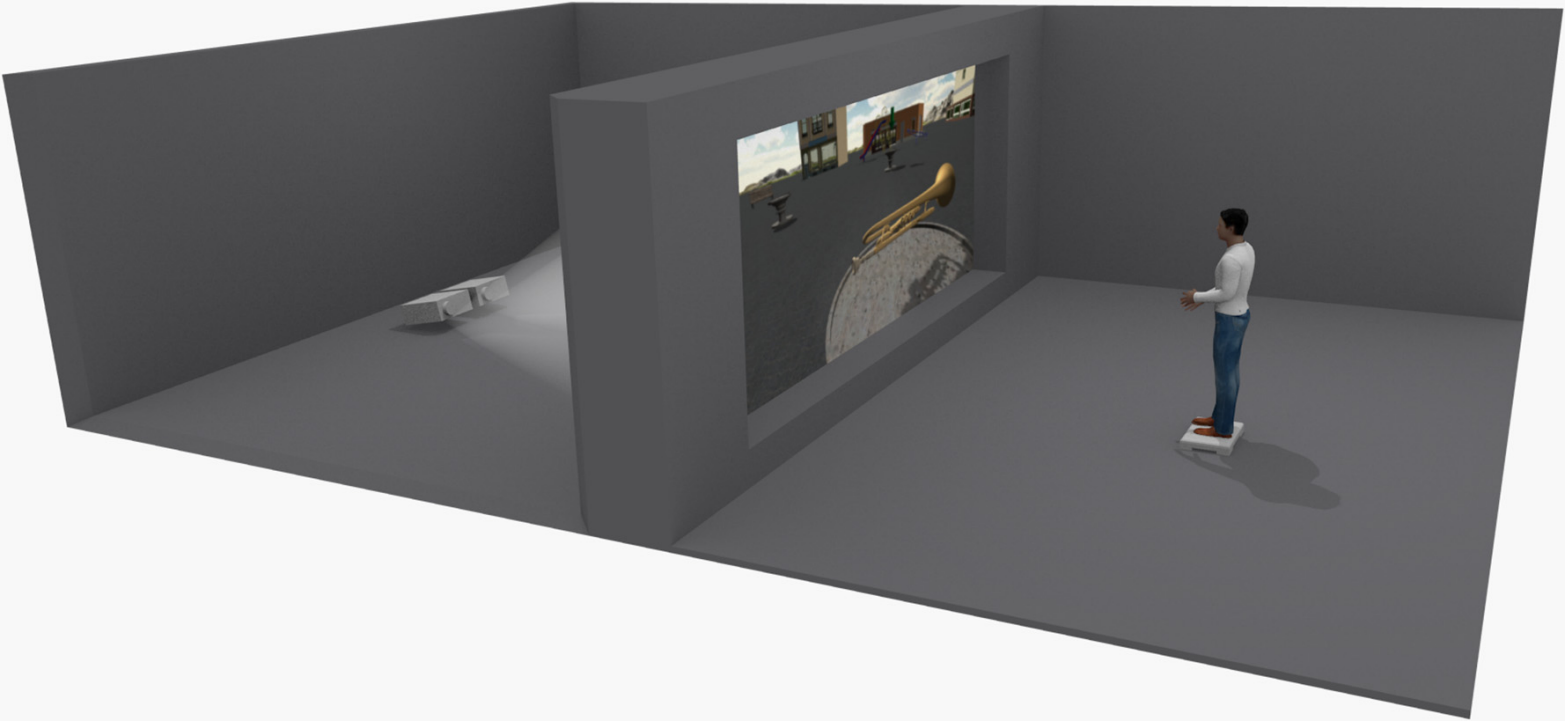
The testing room.

**Fig 9:**
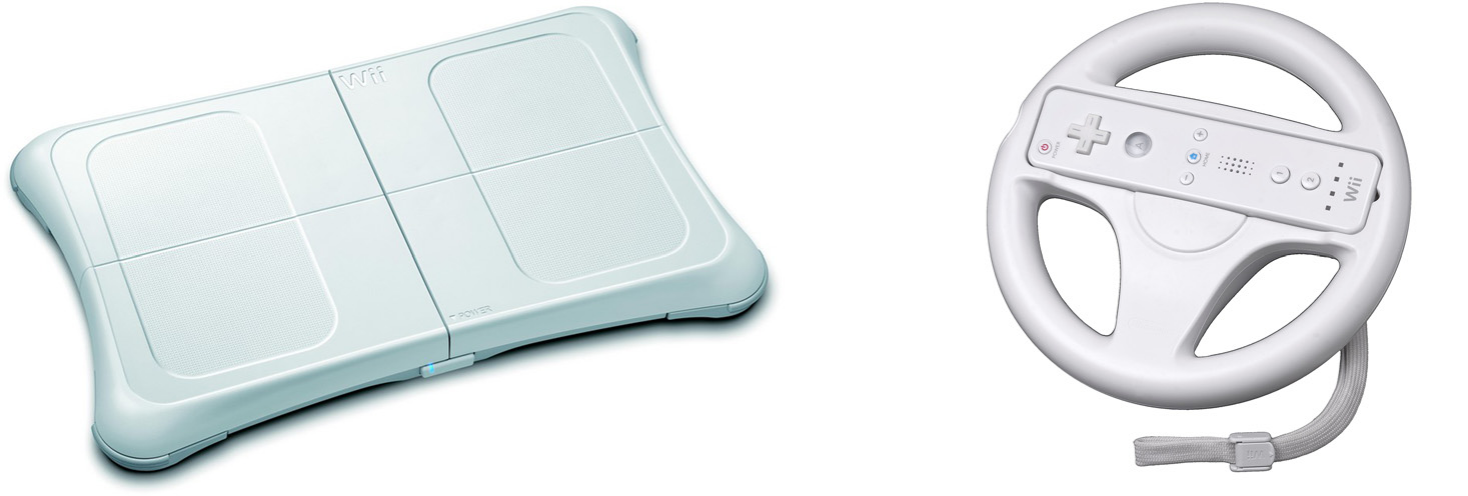
The Wii Balance Board and Wii Mote used to create the NUI interaction.

**Fig 10:**
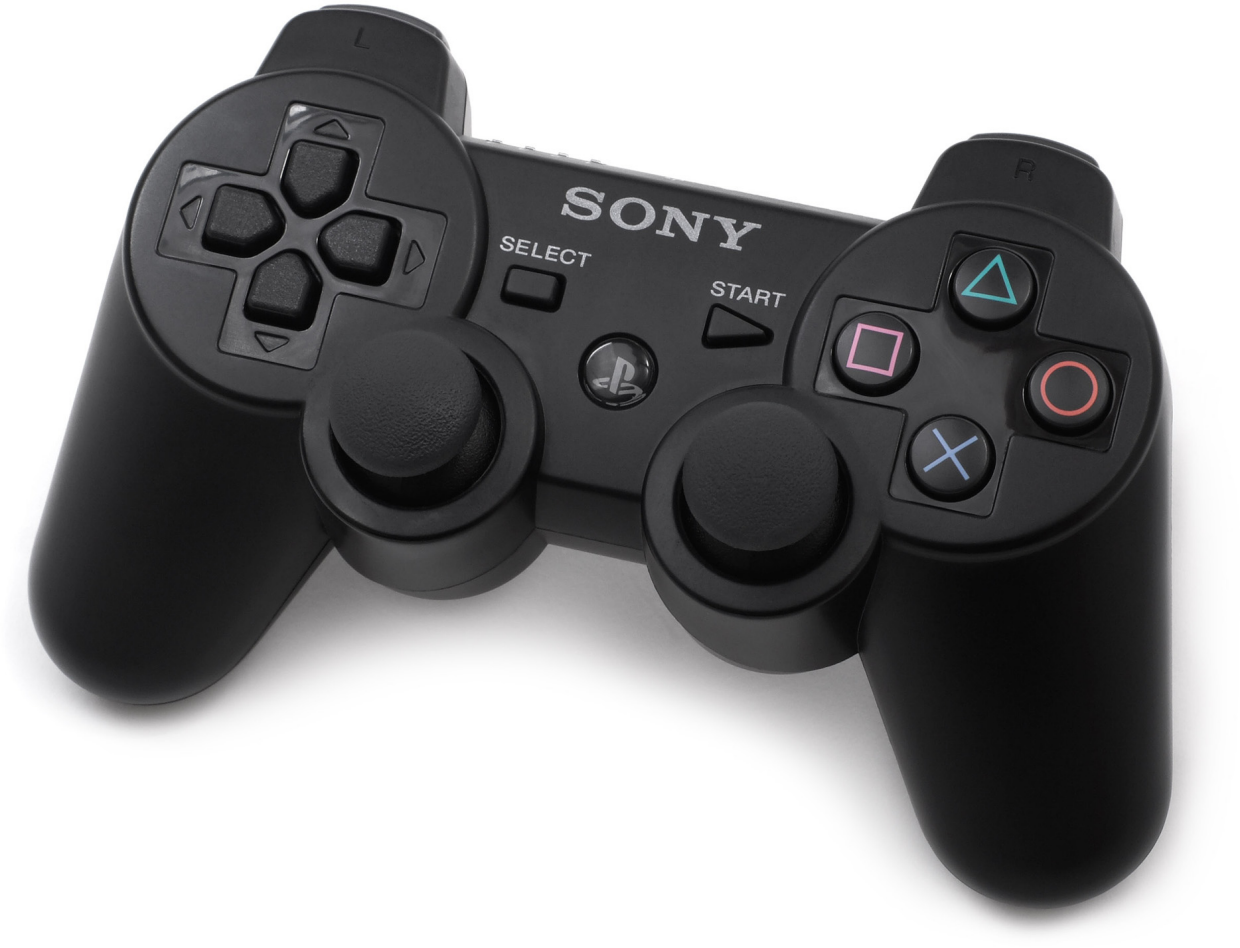
The gamepad used to create the standard interaction.

The following hardware was used:

- The testing room for the task had some special characteristics. First, it was divided into two areas (the projection area and the user area), which were divided by a wall and a translucent screen. The two projectors placed in the projection area project the two images onto the screen. These two images are polarized and a 3D image is created. The user must wear linear polarized 3D glasses in order to see the image correctly. Fig 8 shows a representation of this room.
- Interaction: To develop the user’s interactions, three devices were needed. A Wii Balance Board and a Wii Mote with the wheel accessory were used for the NUI interaction (Fig 9). A “B-Move Gamepad BG Revenge” was used for the standard interface (Fig 10).

## 4. Study

In this section, we describe the participants of the study, the variables considered, and the procedure that the participants followed.

### 4.1 Participants

One-hundred and sixty healthy children, ranging in age from 5 to 10 years old, participated in the study. The mean age was 7.29 ± 1.65 years old. There were 91 boys (56.87%) and 69 girls (43.13%). Table 1 shows the children’s distribution for age and gender. Their parents received written information about the objectives and characteristics of our study and they signed a written consent form on behalf of the children to allow them to participate in it. Also, the children received verbal instructions about what did the study consist at the beginning of the procedure, and where asked whether they might like to participate. This verbal consent was not recorded and the aim was to ensure that all the participants were willing to cooperate. All the participants gave verbal consent. Moreover, all clinical investigation was conducted according to the principles expressed in the Declaration of Helsinki. The Ethics Committee of the Universitat Politècnica de València Spain, approved the study and the written consent form that the parents signed (Reference: 2014–980, approval date: 07/22/2016). The data are available in the S1 File (a Supporting Information File). The participants received a small reward consisting of a diploma right after the testing sessions.

**Table 1:** *Gender and age distribution of the participant*s.

Age	5	6	7	8	9	10	Total
Boys	17	18	20	14	12	10	91
Girls	11	12	15	9	10	12	69

### 4.2 Measurements

For each game of the MnemoCity task, the application stored the following variables in a remote database: the interaction type, the searching and the saving phase times for all of the levels, the table selected in each level, and the score. The table selected in each level corresponded to the place chosen by the child as the one show the object during the searching phase. This variable showed the child’s ability to remember the spatial location of the object. The score of MnemoCity task was the sum of the number of objects placed correctly.

Before starting the task, the users completed the Lang-Stereo-Test [42]. The Lang-Stereo-Test is composed of easy-to-use screening tests that are designed for early detection of problems with stereoscopic vision in children. Two versions of the test plates are available, which only differ in the objects to be recognized. The Lang-Stereo-Test I displays a star, a cat, and a car; the Lang-Stereo-Test II displays a moon, a truck, and an elephant, each of these images have a different disparity. In addition, the Lang-Stereo-Test II contains a star that can be seen by only one eye. Thanks to the Lang-Stereo-Test, an assessment of 3D perception of the children can be performed. There are three possible results: the child sees 3D properly, the child cannot see 3D, and a doubtful result (this means that the child properly recognizes 3D in some of the objects presented, but not all).

Since the sense of presence of the user inside the virtual world is really important in applications of this type [43], we need to know the level of immersion of the user. Therefore, a question about the immersion of the virtual world was included in the Q2 questionnaire. In this questionnaire, we also added some satisfaction and usability questions.

To compare the MnemoCity task with existing assessment procedures, the following two test versions of the Corsi Block-Tapping Task (CBTT) were also used [38]:

- The CBTT (Direct version): The CBTT is a psychological test that assesses visuo-spatial short-term memory. It involves mimicking an evaluator as he/she taps a sequence of up to nine identical spatially separated blocks. The blocks are on a white plastic board that is on top of a table. The evaluator, who points at the blocks is in front of the subject. The sequence starts out simple (usually using two blocks) and becomes more complex until the subject's memory performance diminishes. This number is known as the CBTT Span and averages about 5 for adults [38].
- The CBTT (Reverse version): This is a similar test, the users must not only remember the blocks, and they must also point to them in reverse order. This version assesses the ability to remember and manipulate spatial information and is related to working memory, which is a type of short-term memory that involves the mental manipulation of items.

We considered two variables that are related to performance in the CBTT [38]: the direct scores in each version of the CBTT (*Direct CBTT score* and *Reverse CBTT score* variables); and the number of blocks of the longest sequence that the child can tap correctly (*Direct CBTT span* and *Reverse CBTT span)*.

### 4.3 Procedure

**Fig 11:**
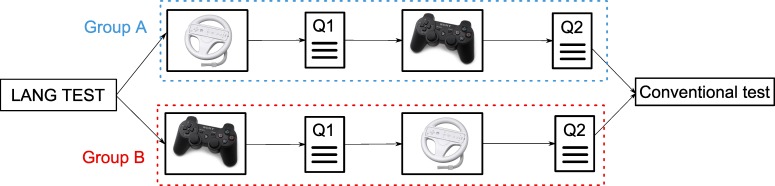
Procedure of the task.

All of the children were randomly assigned to one of two groups based on the interaction used first. At the end of the procedure each child had played the MnemoCity task twice (once for each type of interaction). The different steps of the experimental procedure are shown in Fig 11.

- Group A: This group performed the MnemoCity task first with the NUI interaction and then performed it using the gamepad second.
- Group B: This group performed the MnemoCity task first with the gamepad and then performed it using the NUI interaction second.

When the users had finished playing the task for the first time, they had to fill out the Q1 questionnaire (Table 2). Both groups filled out the Q2 questionnaire after completing the MnemoCity task with the two different interactions (Table 3). Finally, after completing the Q2 questionnaire, the children performed the CBTT (direct and reverse versions) conventionally. The two groups had a similar number of children (72 in Group A and 67 in Group B). The approximate duration to complete the whole procedure was one hour. The test took place Monday through Friday between 9:00 A.M. and 6:00 P.M. Each child was accompanied by a supervisor throughout the entire process.

**Table 2:** Questions of the Q1 questionnaire.

#	Question	Value
US1	Was the game easy to use?	[1.Very difficult / 2.Difficult / 3.Regular / 4.Easy / 5.Very easy]
US2	I always understood what I have to do	[1. Strongly disagree / 2.Disagree / 3.Neither agree nor disagree / 4.Agree / 5.Strongly agree]
SA1	How much fun did you have?	[1. Strongly disagree / 2.Disagree / 3.Neither agree nor disagree / 4.Agree / 5.Strongly agree]
SA2	Would you invite your friends to play the game?	[1. Strongly disagree / 2.Disagree / 3.Neither agree nor disagree / 4.Agree / 5.Strongly agree]
SA3	Would you play this game another time?	[1. Never / 2.Hardly ever / 3.Sometimes / 4. Almost every day / 5.Everyday]
SA4	Score the game from 1 to 5	[1.Very bad / 2.Bad / 3.Regular / 4.Good / 5.Very good]
Q3D	At certain moments the objects came out of the screen	[1. Strongly disagree / 2.Disagree / 3.Neither agree nor disagree / 4.Agree / 5.Strongly agree]

**Table 3:** Questions of the Q2 questionnaire.

#	Question	Value
SA1	How much fun did you have?	[1.Very boring / 2.Boring / 3.Regular / 4.Fun / 5.Very fun]
US1	Was the game easy to use?	[1.Very difficult / 2.Difficult / 3.Regular / 4.Easy / 5.Very easy]
PRE1	Which interaction was more fun?	[1-Wii / 2-Gamepad]
PRE2	Which interaction was easier to use?	[1-Wii / 2-Gamepad]

## 5. Results

A statistical analysis was performed to corroborate our hypotheses. The statistical significance was set at alpha level α = 0.05. The data from the study were analyzed using the statistical open source language and environment for statistical computing and graphics *R* (https://www.r-project.org).

First, data normality was checked with the Levene’s test [44]. Our data did not fit the normal distribution. Therefore, the tests used were non-parametric (Mann-Whitney *U* [45] and Kruskal-Wallis [46] tests).

### 5.1. Lang-Stereo-Test outcomes

The users performed the Lang-Stereo-Test [42] in order to check whether or not they perceived 3D correctly. The results of the 160 users were the following: 139 passed the test correctly, 15 users had doubtful results and 6 users did not pass the test. The correlations on the Lang-Stereo-Test score were analyzed with the MnemoCity score. We performed a Mann-Whitney *U* test (*U* = 754, *Z* = 1.780933, *p* = 0.06, *r* = 0.147391). There was no correlation between these two variables. The means and standard deviation of MnemoCity score indicate that the mean of the users who passed the Lang-Stereo-Test had a better score (the mean of the children who perceived 3D correctly: 2.28±1.27, the mean of the children who did not perceive 3D correctly: 1.67±0.47). We selected the participants that could perceive 3D for the rest of the analyses. Thus, the final sample consisted of 139 children (57% boys and 43% girls). To determine if there were any differences between the users that could perceive 3D correctly and the users that could not perceive 3D correctly, we performed a Kruskal-Wallis test with the following independent variables: Gender, Interaction, Usability, Satisfaction, and MnemoCity Score. The results are shown in Table 4. The Satisfaction variable indicated a statistically significant difference between the two groups. The group that could see 3D correctly showed greater satisfaction than the other group.

**Table 4:** Multifactorial Kruskal-Wallis test results for stereo vision.

Variable	*χ^2^*	df	*p*	Sig.
Gender	1.11	1	0.24	--
Interaction	1.06	1	0.30	--
Usability	6.22	7	0.52	--
Satisfaction	30.57	15	0.01	**
MnemoCity Score	7.03	4	0.14	--

A multifactorial Kruskal-Wallis test was conducted on the influence of five independent variables (Gender, Interaction, Usability, Satisfaction, and MnemoCity Score) for stereo vision.

“**” indicates the statistical significance at level α = 0.05.

“--” indicates that there was no statistical significance.

### 5.2. Interaction outcomes

In order to compare the two interaction types, a Mann-Whitney *U* test was performed to determine whether or not there were statistically significant differences. These results indicate that there were no statistically significant differences regarding the score obtained in the MnemoCity task based on the interaction used (*U* = 2238.5, *Z* = -0.750, *p* = 0.455, *r* = 0.063). We also performed a Mann-Whitney *U* test to find statistically significant differences in gender (*U* = 2471, *Z* = 0.485, *p* = 0.628, *r* = 0.041).

Furthermore, we applied a multifactorial Kruskal-Wallis test (Table 5) with four independent variables (Gender, Usability, Satisfaction, and MnemoCity Score). The Satisfaction variable indicated a statistically significant difference between the users that had the gamepad interaction (M = 9.75, SD = 4.30) and the users that had the natural interaction (M = 10.68, SD = 3.08) in favor of the natural interaction.

**Table 5:** Multifactorial Kruskal-Wallis test results for the type of interaction.

Variable	*χ^2^*	df	*p*	Sig.
Gender	0.04	1	0.847	--
Usability	9.88	6	0.129	--
Satisfaction	40.08	15	< 0.001	**
MnemoCity Score	7.80	4	0.309	--

A multifactorial Kruskal-Wallis test was conducted on the influence of four independent variables (Gender, Usability, Satisfaction, and MnemoCity Score) for the Type of Interaction.

“**” indicates the statistical significance at level α = 0.05.

“--” indicates that there was no statistical significance.

### 5.3. Gender outcomes

A Mann Whitney *U* test was performed to determine if gender affected the MnemoCity score (*U* = 72, *Z* = 0.864, *p* = 0.403, *r* = 0.184). The result indicated that there were no statistically significant differences in gender. Others Mann Whitney U-tests were performed with the classical test scores, regarding gender: the Direct CBTT score (*U* = 48, *Z* = -0.834, *p* = 0.403, *r* = 0.177), and the Reverse CBTT score (*U* = 50, *Z* = -0.702, *p* = 0.516, *r* = 0.149). The results of the tests indicated that there were no statistically significant differences between the performance of boys and girls in the classical method CBTT.

We also applied a multifactorial Kruskal-Wallis test (Table 6) with four independent variables (Interaction, Usability, Satisfaction, and MnemoCity Score). The results show that there were no statistically significant differences regarding gender.

**Table 6:** Multifactorial Kruskal-Wallis test results for gender.

Variable	*χ^2^*	df	*p*	Sig.
Interaction	0.04	1	0.848	--
Usability	12.42	6	0.050	--
Satisfaction	15.06	15	0.448	--
MnemoCity Score	1.68	4	0.796	--

A multifactorial Kruskal-Wallis test was conducted on the influence of four independent variables (Interaction, Usability, Satisfaction, and MnemoCity Score) for gender.

“**” indicates the statistical significance at level α = 0.05.

“--” indicates that there was no statistical significance.

### 5.4. Preference and depth perception outcomes

In the Q2 questionnaire, two questions (PRE1-PRE2) about the preference of the interaction were included to determine which of the two interactions types the users preferred. Fifty-one percent of the users preferred the Wii interaction, and the rest (49%) preferred the gamepad interaction. With regard to the ease of use, 38% of the users thought that the Wii interaction was easier, and the rest (62%) thought that the gamepad interaction was easier. Two tests were performed to determine whether or not there were differences regarding the preference questions between the two groups. The results are shown in Table 7.

**Table 7:** Interaction and 3D preferences.

Variable	Group A	Group B	*U*	*Z*	*p*-value	*r*	Sig.
PRE1-Which interaction was more fun?	1.54±0.50	1.44±0.50	3605	1.26	0.214	0.10	--
PRE2-Which interaction was easier to use?	1.56±0.50	1.66±0.50	2924	-1.41	0.175	0.11	--
Q3D- At certain moments, the objects came out of the screen	3.59±1.34	3.75±1.30	3052	-0.79	0.431	0.06	--

Mann-Whitney U tests about the preferences. Group A is the one where the users played with the NUI first, and Group B is the one where the users played with the gamepad first. The PRE1 and PRE2 questions have two options (1: NUI and 2: Gamepad). The Q3D question represents the opinion of the user about the 3D sensation of the task.

“**” indicates the statistical significance at level α = 0.05.

“--” indicates that there was no statistical significance.

In the Q1 questionnaire, there was a question (Q3D) about the depth perception. The question had a high score 3.6 (1–5 scale). We also performed a multifactorial Kruskal-Wallis test (Table 8) with five independent variables (Gender, Type of Interaction, Usability, Satisfaction, and MnemoCity Score). The results show that there were no statistically significant differences.

**Table 8:** Multifactorial Kruskal-Wallis test results for depth perception.

Variable	*χ^2^*	df	*p*	Sig.
Gender	0.45	1	0.503	--
Interaction	0.47	1	0.495	--
Usability	7.24	7	0.404	--
Satisfaction	14.06	14	0.450	--
MnemoCity Score	3.53	4	0.473	--

A multifactorial Kruskal-Wallis test was conducted on the influence of four independent variables (Gender, Interaction, Usability, Satisfaction, and MnemoCity Score) for depth perception.

“**” indicates the statistical significance at level α = 0.05.

“--” indicates that there was no statistical significance.

### 5.5. Short-term memory outcomes

**Fig 12:**
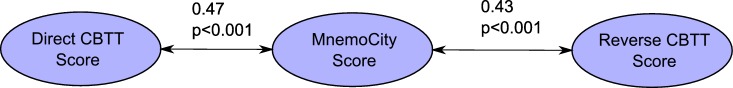
The Correlation plot.

**Fig 13:**
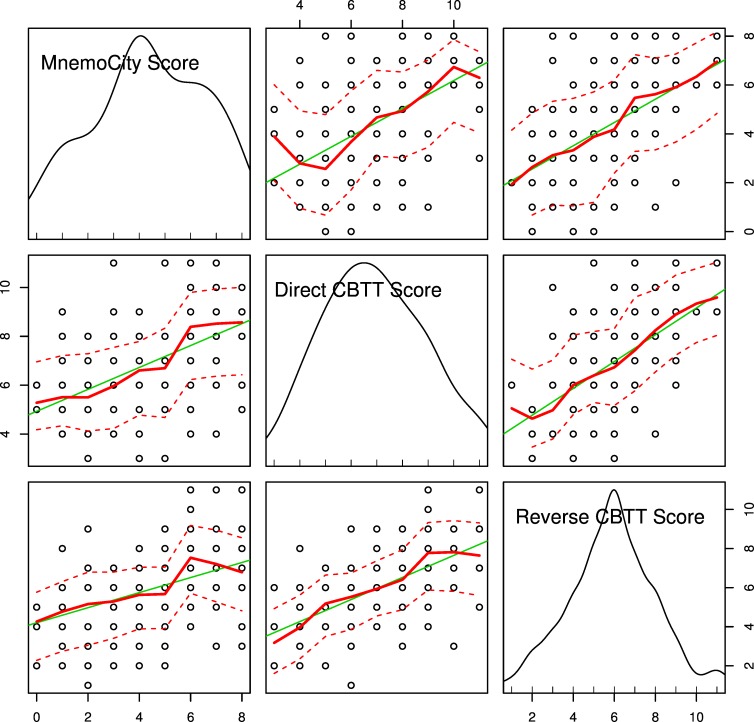
The Matrix plot for correlations between traditional tests and the MnemoCity task. The distributions of the variables show the MnemoCity Score, the Direct CBTT Score, and the Reverse CBTT Score. The interactions between the three variables are shown in the other plots of the matrix. The red lines represent the mean and the standard deviation for each value, and the green lines represent the regression line of the correlation.

The MnemoCity score variable is a measure of short-term memory capability. The MnemoCity score can have a value between zero and four, based on the number of correct responses. Table 9 presents a descriptive analysis of this variable taking into account the independent variables (Age, Gender, and Type of interaction). As can be observed in Table 9, the task was not easy to learn for the younger children. They were not able to remember the location of all of the objects trained. Even, some of the older children did not perform the task perfectly. To compare the MnemoCity task performance level with the performance level obtained in traditional methods (the Direct and Reverse versions of the CBTT), we calculated their correlations. We did these correlations with the entire sample of 139 children. The Spearman correlation was used, and the results are shown in Fig 12. The Spearman correlation effect size was rho (ρ). The MnemoCity score was correlated with the Direct CBTT score (ρ = 0.47, *p* < 0.001**) and the Reverse CBTT score (ρ = 0.43, *p* < 0.001**). The correlation between the MnemoCity score and the two traditional test scores (Direct and Reverse CBTT score) are shown graphically in Fig 13. It can be observed that the correlations are positive and linear in all cases.

**Table 9:** Descriptive data of the MnemoCity score.

Variable	Value	Mean	Standard deviation
**Age**	5 years old	1.10	± 0.81
	6 years old	1.62	± 1.03
	7 years old	2.09	± 1.25
	8 years old	2.91	± 0.90
	9 years old	3.10	± 0.99
	10 years old	3.24	± 0.81
**Gender**	Girls	2.33	± 1.25
	Boys	2.20	± 1.27
**Type of interaction**	Gamepad	2.34	± 1.36
	NUI	2.21	± 1.17

The descriptive data of the independent variables (Age, Gender, and Type of interaction) related to the task variable for evaluating short-term memory (MnemoCity score).

### 5.6. Usability and satisfaction outcomes

The children answered two questions about usability and four questions about satisfaction with the MnemoCity task. All of these questions were answered in the Q1 questionnaire. In the Q2 questionnaire, the children only answered SA1 and US1. A Mann-Whitney U test for each question was performed to determine if there was any difference in the answers between the two groups. No statistically significant differences were found between the two groups. The results are shown in Table 10.

**Table 10:** Usability and satisfaction questions.

Question	Group A	Group B	*U*	*Z*	*p*-value	*r*	Sig.
US1- Was the game easy to use?	4.04±0.94	4.25±0.90	2837	-1.59	0.113	0.13	--
US2- I always understand what I have to do	4.73±0.52	4.65±0.73	3360	0.36	0.713	0.03	--
SA1- How much fun did you have?	4.65±0.48	4.64±0.62	3150	-0.53	0.632	0.04	--
SA2- I would invite my friends to play this game	4.16±0.92	4.16±1.11	3085	-0.70	0.479	0.06	--
SA3- I Would play this game another time	3.89±1.01	4.04±0.99	2997	-1.01	0.317	0.08	--
SA4- Score the game from 1 to 5	4.46±0.75	4.54±0.69	3131	-0.58	0.567	0.05	--

Mann-Whitney U tests for the satisfaction and usability questions. Group A is the one where the users played with the NUI first, and Group B is the one where the users played with the gamepad first. All the questions are in a Likert scale (1–5).

“**” indicates the statistical significance at level α = 0.05.

“--” indicates that there was no statistical significance.

The users answered the SA1 and US1 question twice. Since the children answered these two questions twice, we can verify whether they changed their opinion about the first and second interaction, as they answered the two questions twice (Table 11). The only difference obtained was in the question "*Was the game easy to use*?" and it seems that users who played with the gamepad first found it more difficult to play the game with the Wii second.

**Table 11:** Interaction preferences.

Group	Question	Q1 questionnaire	Q2 questionnaire	*W*	*Z*	*p*-value	*r*	Sig.
**Group A**	SA1- How much fun did you have?	4.65±0.48	4.59±0.73	3311	-0.20	0.875	0.02	--
	US1- Was the game easy to use?	4.04±0.94	4.05±1.05	3259	-0.36	0.724	0.03	--
**Group B**	SA1- How much fun did you have?	4.64±0.62	4.62±0.64	3228	0.12	0.907	0.01	--
	US1- Was the game easy to use?	4.25±0.90	3.77±1.22	3880	2.46	0.014	0.20	**

Wilcoxon Singed-rank tests for the repeated questions in the Q1 and Q2 questionnaires. This table shows the differences between the views of users about how fun the game was and how easy the game was to use.

“**” indicates the statistical significance at level α = 0.05.

“--” indicates that there was no statistical significance.

The general values of satisfaction and usability obtained from the questions were calculated by summing all of the values of the question and obtaining their means for usability (US1 and US2) and for satisfaction (SA1, SA2, SA3, and SA4). The children scored the task with an average of 3.49 over 5. And the usability obtained was 4.26 over 5.

## 6. Discussion

In this work, the capability of our MnemoCity task was tested to assess spatial short-term memory in children from 5 to 10 years old. Some applications for assessing spatial memory in humans have been developed previously [8–10,22]. These applications used basic methods of human computer interaction that could interfere with the quality of the user's immersion in the virtual environment. The quality of the immersion could affect the correct performance of the tasks [13,14]. Moreover, these tasks are designed to be performed by adults and not by children. A review of the literature indicates that a task that incorporates stereoscopy for the assessment of spatial short-term memory has not yet been developed. In our work, we have created a task that uses Natural User Interfaces and a large stereoscopic screen to facilitate immersion and interaction.

There have been few attempts to address spatial short-term memory through experimental tasks involving the movement of a child around an environment. The study by Smith et al. [47] presented a searching task for target locations that were hidden under a 7×7 grid. In this task, the goal was to probe the search efficiency of the child. Piccardi et al. [48] studied short-term memory in children with the use of the Walking Corsi Test (WalCT). This test was a larger version of the CBTT [38] with a surface area of 2.5×3 meters. The child had to reproduce a walking sequence of the white points on the floor. In one of our previous works (Juan et al. [6]), we developed a task that incorporated augmented reality to evaluate spatial short-term memory in children. In this case, the task mixed virtual elements with the real world. Virtual elements were the objects to remember. The real elements in this work were the place and the strategically placed boxes throughout the room. The virtual elements were the objects inside the boxes. The benefit of using augmented reality was the possibility to have multiple objects that are not limited to the real world and the possibility to control the showing time of the objects. This is important because the codification time is essential in a spatial memory task. The users scored the task with high values of satisfaction and usability. Taking into account all of the above-mentioned features (virtual reality, natural user interaction, and stereoscopy), to our knowledge, this is the first time that a task of these characteristics has been presented for this purpose.

The Lang-Stereo-Test [49] that was applied to our sample showed that 87% of the children could correctly perceive 3D and 13% could not perceive 3D properly. This result is consistent with other studies that indicated that between 5% and 10% of the population do not have stereo vision [49,50]. Furthermore, the Kruskal-Wallis test corresponding to stereo vision (Table 4) showed that the users that perceive 3D properly have greater satisfaction when performing the task. This may be due to the fact that the application was especially designed for 3D perception of the virtual environment and the participants that cannot see 3D properly enjoy the task less than the others.

A comparison between our task with a commonly used task in neuropsychological assessment, the CBTT [38], demonstrated that the performance of our task is related to short-term memory ability in children. The results of correlation tests indicated that the MnemoCity task shows validity for the assessment of spatial memory in children. The correlation of this task with the CBTT [38], (Direct and Reverse CBTT) is significant. The correlation was average in both cases, with a value of 0.44 and 0.42, respectively. This difference may be due to the fact that children handle a different type and form of information. In the CBTT the participant is in front of an evaluator and the participant remains in the same place throughout the entire process. In the MnemoCity task, the user is moving and seeing objects in the same way as occurs in their daily life. Therefore, the two tasks share some components of spatial short term memory, even though the procedure and features of the CBTT and the MnemoCity task are significantly different. The correlation of Direct and Reverse CBTT scores and the MnemoCity score was linear dependent, as shown in the Fig 13. To our knowledge, this is the first time that a virtual immersive environment has been compared directly with traditional methods for the assessment of short-term memory in children.

It should be pointed out that there is no real movement of the subject, since the participants used natural interaction to explore the virtual room. Recent studies have indicated that there are differences in the dynamics of acquisition of landmark-referenced (allocentric) knowledge relative to view-referenced (egocentric) knowledge [51]. In our task, at the end of each search phase, the child returns to the center of the scene, and the virtual world is rotated 180° from the original position before starting the second phase. This means that the egocentric components of navigation did not provide any useful information and only the allocentric reference frame is valid for an adequate orientation. Previous studies [52–54] have demonstrated that some short-term memories are based on egocentric components of navigation. In the work by Wang et al. [52], the users are disorientated by pivoting turns. After the disorientation, the egocentric component was eliminated from the navigation. In our task, using a virtual environment allows us to eliminate egocentric information in a way that is less annoying to the user.

With regard to user interaction, in the design phase of the MnemoCity task, we tried to find an interface that was as unobtrusive as possible so that the user could focus on the task. We also included walking motion to achieve a more realistic experience for the user while he/she was exploring the virtual environment. For this reason, we opted for a Natural User Interface. We also wanted to verify the advantages of using NUI compared to using a standard and passive motion-based interaction. Our study has shown that the use of the standard interface (gamepad) did not differ significantly for the usability and satisfaction questions or for the assessment of the task. Our explanation for this result is that most children are ‘digital natives’ and are already familiar with standard interaction methods. Thanks to the incorporation of Wii and Kinect, more and more children have also become familiar with NUI interaction. Moreover, the interaction adaptation level helped the children in our study to get used to our interface. Therefore, their mastery of current trends, their ability to adapt to any technological change, and the different levels of our task have all contributed to making the two types of interaction were less noticeable for them. To our knowledge, previous comparisons among interaction methods in other studies (e.g., [26, 27]) have not carried out a comparison like the one proposed in this paper. The only difference between the two interactions was the preference in the *PRE2* question *“Which interaction was easier to use*?*”*. A total of 62% of the users thought that the gamepad was easier to use. Despite the differences between our study and Rauterberg’s study [26], it can be observed that there is a similarity between their results and ours. In Rauterberg’s study, it was found that the users rated touch and mouse interactions as being easier than the custom-made Digital Playing Desk, and, in our work, the users thought that the gamepad was easier than the NUI. Our explanation for this result is that even though interaction with the NUI is more natural, users must be aware of what they are doing with their feet and hands, whereas with the gamepad they only have to think about what they are doing with their hands. Finally, as mentioned above, the type of interaction did not affect the final score for the MnemoCity task. Although unexpected, this is a good result because it means that the task is well suited for the assessment of spatial memory and that the two interaction types can be used for this purpose. Consequently, this application can also be used by children with reduced mobility [17,55].

With regard to gender differences in the MnemoCity score, the results indicated that there were no statistically significant differences for gender. Also, the classical task CBTT [38] did not show any statistically significant differences. The similar performance between genders may be supported by other studies in which there were no statistically significant differences in gender when the spatial memory was tested on children [6,56–58]. Therefore, our results regarding gender are in line with the conclusions reached by those works and corroborate our third hypothesis (there were no statistical significant differences for the performance of the task between genders). Other studies have shown differences between genders. For example, Moffat et al. [19] found male superiority in a study with VR environments that were developed to test the egocentric spatial orientation of adult participants. The egocentric orientation is based on one’s body position in space (i.e., idiothetic information). In our task, the idiothetic information was irrelevant for the children because the virtual world was rotated before the testing phase. Therefore, the environmental cues and their arrangement in space were very important in helping children to locate the correct place of the object (i.e., allocentric information). The different navigational strategies promoted in each task could determine the existence or absence of gender differences, as was previously suggested [59]. The fact that the MnemoCity task has a low level of difficulty could explain the absence of differences between boys and girls.

## 7. Conclusions

The MnemoCity task, which incorporates stereoscopy, virtual environments, and NUI has been developed to assess spatial short-term memory in children. This task assessed children's ability to retain the position of the objects as a way to test spatial short-term memory in a natural environment. One of the main advantages of our system is that it allows the user to feel immersed in a large-scale complex virtual environment, which gives the user the sensation of being in a real environment. These sensations are not possible using traditional procedures or more basic visualization devices. Even though the system allows motion from a user-centered perspective and proves the navigational competence ecologically, the system assesses the user’s short-term spatial memory in a controlled manner (i.e., it allows the user’s position to be controlled to prevent an egocentric strategy). In addition, automatically stores information about the user’s performance. Automatic storage of information is not possible with traditional procedures. Finally, another advantage of the system is that participation by an expert psychologist is only necessary in the design phase of the system; the task could be performed without the physical presence of an expert psychologist. The MnemoCity task could be used with more common 3D visualization devices such as HMDs (e.g., Oculus Rift). Since the interaction method did not significantly influence the assessment of short-term memory, the MnemoCity task could also be used with more traditional input devices. The MnemoCity task performance was compared with traditional neuropsychological procedures for the assessment of spatial short-term memory, and the usability and satisfaction of the participants were measured. The scores of the traditional procedures were correlated with the MnemoCity score. This means that the developed task could be used as an entertaining method to assess or train children in spatial short-term memory skills from an ecological assessment perspective, since the two types of interactions have shown that they can assess spatial memory. The MnemoCity task can potentially be used as part of an evaluation in children with and without motor problems. As future work, the possibility of using this task for the assessment of short-term spatial memory and other cognitive abilities related to spatial processing can be considered. We would also like to test this task in populations with special education needs, learning disabilities, or situations where cognitive impairment would likely affect spatial orientation or memory skills.

## Supporting Information

S1 File Raw data file. Data used in the analysis.
(XLSX)
